# Systemic lipolysis promotes physiological fitness in *Drosophila melanogaster*

**DOI:** 10.18632/aging.204251

**Published:** 2022-08-30

**Authors:** Linshan Shang, Elizabeth Aughey, Huiseon Kim, Timothy D. Heden, Lu Wang, Charles P. Najt, Nicholas Esch, Sophia Brunko, Juan E. Abrahante, Marissa Macchietto, Mara T. Mashek, Todd Fairbanks, Daniel E. L. Promislow, Thomas P. Neufeld, Douglas G. Mashek

**Affiliations:** 1 Department of Biochemistry, Molecular Biology and Biophysics, University of Minnesota, Minneapolis, MN 55455, USA; 2 Department of Genetics, Cell Biology, and Development, University of Minnesota, Minneapolis, MN 55455, USA; 3 Department of Environmental and Occupational Health Sciences, University of Washington, Seattle, WA 98105, USA; 4 University of Minnesota Informatics Institute, Minneapolis, MN 55455, USA; 5 Minnesota Supercomputing Institute, University of Minnesota, Minneapolis, MN 55455, USA; 6 Department of Biology, University of Washington, Seattle, WA 98195, USA; 7 Department of Lab Medicine and Pathology, University of Washington School of Medicine, Seattle, WA 98195, USA; 8 Department of Medicine, Division of Diabetes, Endocrinology and Metabolism, University of Minnesota, Minneapolis, MN 55455, USA

**Keywords:** *brummer*, lipolysis, physiological fitness, stress resistance, proteostasis

## Abstract

Since interventions such as caloric restriction or fasting robustly promote lipid catabolism and improve aging-related phenotypical markers, we investigated the direct effect of increased lipid catabolism via overexpression of *bmm* (*brummer*, FBgn0036449), the major triglyceride hydrolase in *Drosophila,* on lifespan and physiological fitness. Comprehensive characterization was carried out using RNA-seq, lipidomics and metabolomics analysis. Global overexpression of *bmm* strongly promoted numerous markers of physiological fitness, including increased female fecundity, fertility maintenance, preserved locomotion activity, increased mitochondrial biogenesis and oxidative metabolism. Increased *bmm* robustly upregulated the heat shock protein 70 (Hsp70) family of proteins, which equipped the flies with higher resistance to heat, cold, and ER stress via improved proteostasis. Despite improved physiological fitness, *bmm* overexpression did not extend lifespan. Taken together, these data show that *bmm* overexpression has broad beneficial effects on physiological fitness, but these effects did not impact lifespan.

## INTRODUCTION

A large body of literature shows that lipid metabolism exerts profound regulatory effects on aging and affects stress responses [1]. Moreover, growing evidence shows that interventions that promote lipid catabolism increase lifespan [2, 3]. For example, the induction of the lysosomal lipase LIPL-4 in *C. elegans* increases mitochondrial β-oxidation to reduce lipid storage and promote longevity [4]. In *Drosophila*, overexpression of genes involved in fatty acid β-oxidation extends lifespan and enhances stress tolerance related to the *Drosophila* forkhead transcription factor (dFOXO) activation [5–7]. The protein abundance of ATGL, the primary triacylglycerol (TAG) lipase in mammals, and hormone-sensitive lipase are reduced in adipose tissue of aged (24-month-old) C57BL/6J mice compared with young mice [8]. Adipose expression of ATGL, FOXO1 and the β2-adrenergic receptor, all of which contribute to lipolysis, are reduced in both 14 and 18 month old C57BL/6J mice [9]. ATGL is also decreased in skeletal muscle of old mice [10]. These studies are consistent with reports of reduced lipolysis in adipose tissue of aged mice [11] and humans [12].

The importance of lipolysis by ATGL is highlighted by the fact that ATGL-deficient mice die prematurely due to cardiac lipid accumulation and cardiomyopathy [13]. In contrast, overexpression of ATGL-1 (the *C. elegans* homologue of ATGL) increases *C. elegans* lifespan and the life-extending effects of dietary restriction (DR) are blocked by ATGL-1 disruption [14]. In *Drosophila,* BMM is the well-characterized triglyceride lipase and mediates lipid catabolism through conserved signaling pathways [15, 16]. *bmm* overexpression decreases lipid droplet size, while *bmm*-deficiency augments TAG storage, causes obesity, and shortens lifespan, demonstrating an important role in maintaining lipid homeostasis in *Drosophila*. However, the effects of overexpression of *bmm* on *Drosophila* physiological fitness and lifespan remain to be explored. Since health-promoting interventions such as DR, exercise, and fasting are all known to promote lipolysis, we carried out comprehensive characterization of systemically increased lipolysis and tested the role of lipolysis in regulating physiological parameters in *Drosophila*.

## RESULTS

### Global *bmm* overexpression effectively promotes lipolysis in *Drosophila*

**Figure 1:**
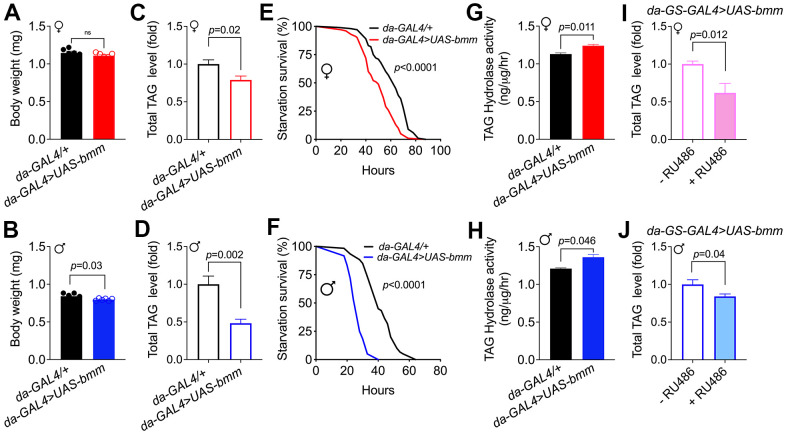
**Global *bmm* overexpression effectively promotes lipolysis in *Drosophila*.** (**A**, **B**) Body weight of *da-GAL4>UAS-bmm* flies with ubiquitous *bmm* overexpression compared with control *da-GAL4/+* flies. (**C**, **D**) TAG content in *da-GAL4/+* and *da-GAL4>UAS-bmm* flies. (**E**, **F**) The survival curves of *da-GAL4/+* vs. *da-GAL4>UAS-bmm* flies under starvation conditions fed on 1.5% w/v agar as a water source. n=100 for female group, n=60 for male group, and *p* value was determined by log-rank analysis. (**G**, **H**) TAG hydrolase activity of *da-GAL4>UAS-bmm* flies compared with control *da-GAL4/+* flies. (**I**, **J**) TAG content in inducible *da-GS-GAL4>UAS-bmm* flies with or without 50 μM RU486 induction. n=6 replicates and each replicate contained 7-10 flies for (**A**–**D**, **I**, **J**); n=3 replicates and each replicate contained 20 flies for (**G**, **H**). Data are shown as mean±SEM and analyzed by two-tailed Student *t*-test. See also Supplementary Figure 1.

To promote lipolysis across tissue in *Drosophila*, we overexpressed *bmm* ubiquitously using a constitutive *daughterless*-*GAL4* driver crossed with a *UAS-bmm* line [15]. No significant body weight difference was observed in female *bmm* overexpression flies *versus* controls, while *bmm* overexpression male flies showed ~5% body weight decrease (*p*=0.03) (Figure 1A, 1B). Consistent with the function of BMM in TAG Consistent with the function of BMM in TAG catabolism, total TAG levels were decreased in both female and male *bmm* overexpression flies compared to their controls (Figure 1C, 1D). When subjected to starvation, flies with overexpression of *bmm* died faster than control flies (Figure 1E, 1F), which likely resulted from reduced energy storage. The increased triglyceride hydrolase activity (Figure 1G, 1H) in both female and male *bmm* overexpression flies is consistent with the known effects of BMM and the reduced TAG levels. To gain insights into the effects of *bmm* overexpression on lipid profiles, we carried out a comprehensive targeted lipidomic analysis. We observed broad decreases in TAG content across TAG lipid species in both female and male flies with *bmm* overexpression (Supplementary Figure 1A, 1B). We also detected effects of *bmm* overexpression on other lipid species, including an increase in various phospholipids and a decrease in free fatty acids in both sexes except for increased long chain saturated fatty acids in males (Supplementary Figure 1C, 1D). We also used the inducible GeneSwitch (GS) system to investigate the effects of *bmm* overexpression. The GeneSwitch system allows temporal control of gene expression by using a modified GAL4 protein that is active only when the synthetic progesterone analogue (mifepristone, RU486) is administered to the flies [17]. Using the global inducible *daughterless-GS-GAL4* line, we investigated the effects of *bmm* overexpression specifically in adulthood by feeding the flies RU486 after eclosion. Similar to non-inducible lines, total TAG level was significantly decreased in both female and male *bmm* overexpression flies compared to their controls (Figure 1I, 1J). No significant differences of TAG level were detected in control flies under vehicle and RU486 induction conditions (Supplementary Figure 1E, 1F). In addition, *bmm* overexpression increased food intake compared with controls in female flies, while male flies showed no significant food intake differences (Supplementary Figure 1G, 1H), suggesting that the decreased TAG levels were not due to reduced caloric intake. Overall, these data suggest that *bmm* overexpression promotes lipid catabolism and reduces lipid storage using both non-inducible and inducible genetic drivers.

### *bmm* overexpression promotes physiological fitness in both female and male *Drosophila*

**Figure 2:**
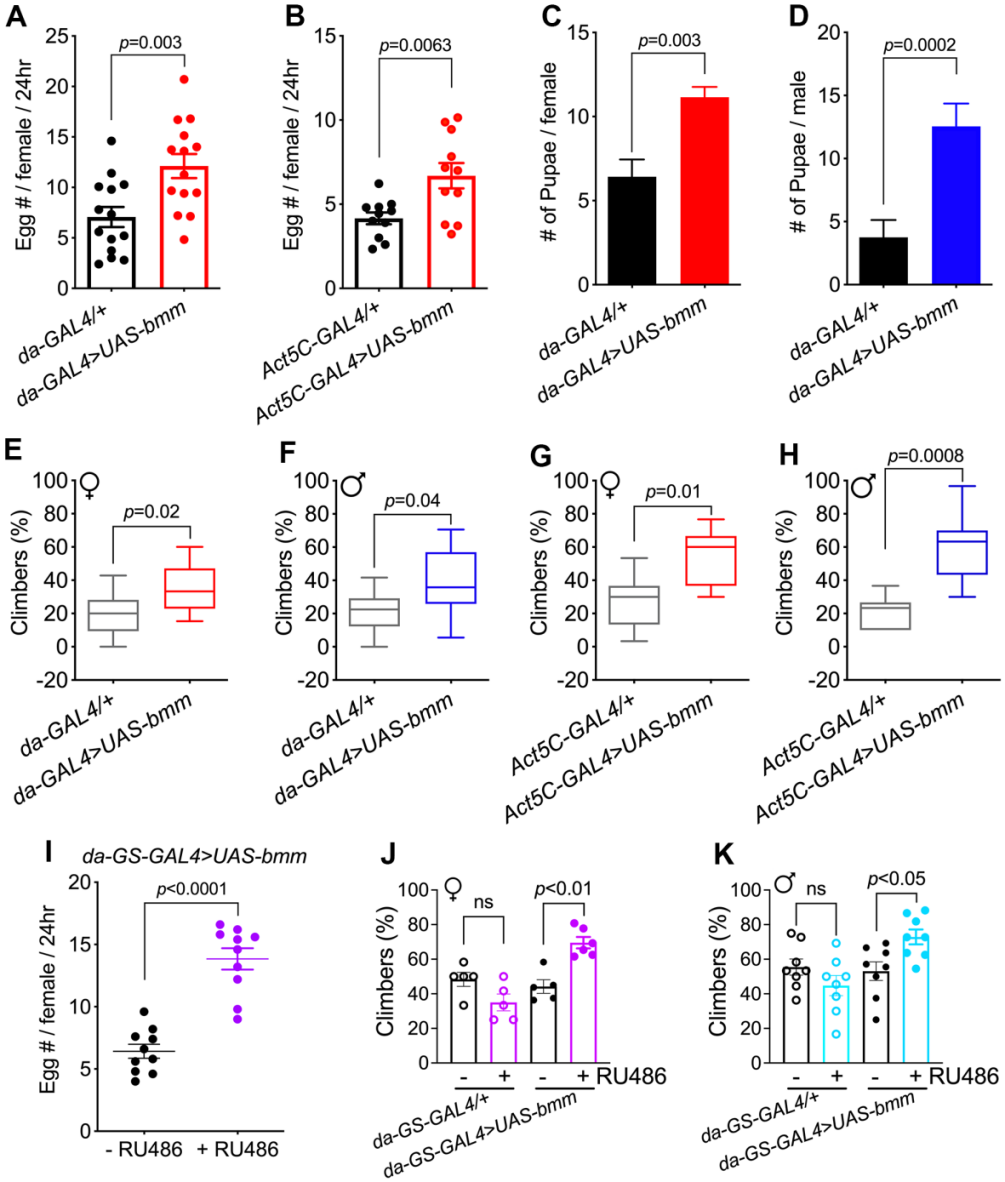
***bmm* overexpression promotes physiological fitness in both female and male *Drosophila*.** (**A**) Fecundity of 2-week-old *da-GAL4/+* vs. *da-GAL4>UAS-bmm* female flies. n=168 for *da-GAL4/+* and n=154 for *da-GAL4>UAS-bmm* group. (**B**) Fecundity of 2-week-old *Act5C-GAL4/+* vs. *Act5C-GAL4>UAS-bmm* female flies. n=93 for *Act5C-GAL4/+* and n=84 for *Act5C-GAL4>UAS-bmm* group. (**C**) Number of pupae produced by *da-GAL4/+* vs. *da-GAL4>UAS-bmm* female flies at 28 days of age paired with young male *w^1118^* flies. n=25 for each genotype. (**D**) Number of pupae produced by *w^1118^* virgin female flies after paired with *da-GAL4/+* or *da-GAL4>UAS-bmm* male flies at 30 days of age. n=80 for each genotype. Data are shown as mean±SEM and analyzed by two-tailed Student *t*-test in (**A**–**D**). (**E**) Locomotion analysis of female *da-GAL4/+* vs. *da-GAL4>UAS-bmm* flies at 38 days of age. n=84-151 for each group. (**F**) Locomotion analysis of male *da-GAL4/+* vs. *da-GAL4>UAS-bmm* flies at 40 days of age. n=147-172 for each group. (**G**, **H**) Locomotion analysis of *Act5C-GAL4/+* vs. *Act5C-GAL4>UAS-bmm* at 35 days of age. n=105 for each group. Data are shown as box and whisker plot and analyzed by two-tailed Student *t*-test in (**E**–**H**). (**I**) Fecundity of 2-week-old inducible *da-GS-GAL4>UAS-bmm* female flies with or without RU486 induction. n=50 for each group. Data are shown as mean±SEM and analyzed by two-tailed Student *t*-test. (**J**) Locomotion analysis of female flies using inducible *da-GS-GAL4* driver at 76 days of age with or without RU486 induction. n=60-73 for each group. (**K**) Locomotion analysis of male flies using inducible *da-GS-GAL4* driver at 72 days of age with or without RU486 induction. n=95-109 for each group. Data are shown as mean±SEM and analyzed by one-way ANOVA in (**J**, **K**). See also Supplementary Figure 2.

We explored the effects of ubiquitously overexpressed *bmm* in flies by investigating numerous physiological markers. Compared with control *da-GAL4/+* flies, *da-GAL4>UAS-bmm* flies had significantly increased fecundity (Figure 2A). Increased egg laying capacity was also observed when compared with *+/UAS-bmm* flies (Supplementary Figure 2A). This enhanced fecundity was also observed in *bmm* overexpression female flies using a different constitutive *Act5C-GAL4* driver (Figure 2B). Moreover, aged female and male flies with *bmm* overexpression maintained active fertility as indicated by a significantly higher number of produced pupae (Figure 2C, 2D).

Reduced mobility is a conserved hallmark of aging, thus, we performed negative geotaxis assays to evaluate the effects of *bmm* on locomotion. In young flies, similar locomotion was observed between control and *bmm* overexpression flies. When flies reached older age, both female and male *bmm* overexpression flies exhibited increased locomotion compared with control flies (Figure 2E–2H and Supplementary Figure 2B, 2C). Similar benefits including increased egg laying and preserved climbing capacity were observed using the inducible *da-GS-GAL4* to drive *bmm* expression (Figure 2I–2K). No differences were observed in fecundity and locomotion activity in control *da-GS-GAL4/+* flies treated with vehicle or RU486 (Supplementary Figure 2D and Figure 2J, 2K). These data indicate that *bmm* overexpression preserves fecundity and mobility in older flies.

### *bmm* overexpression shows enhanced resistance upon most stress challenges and improves protein homeostasis

**Figure 3:**
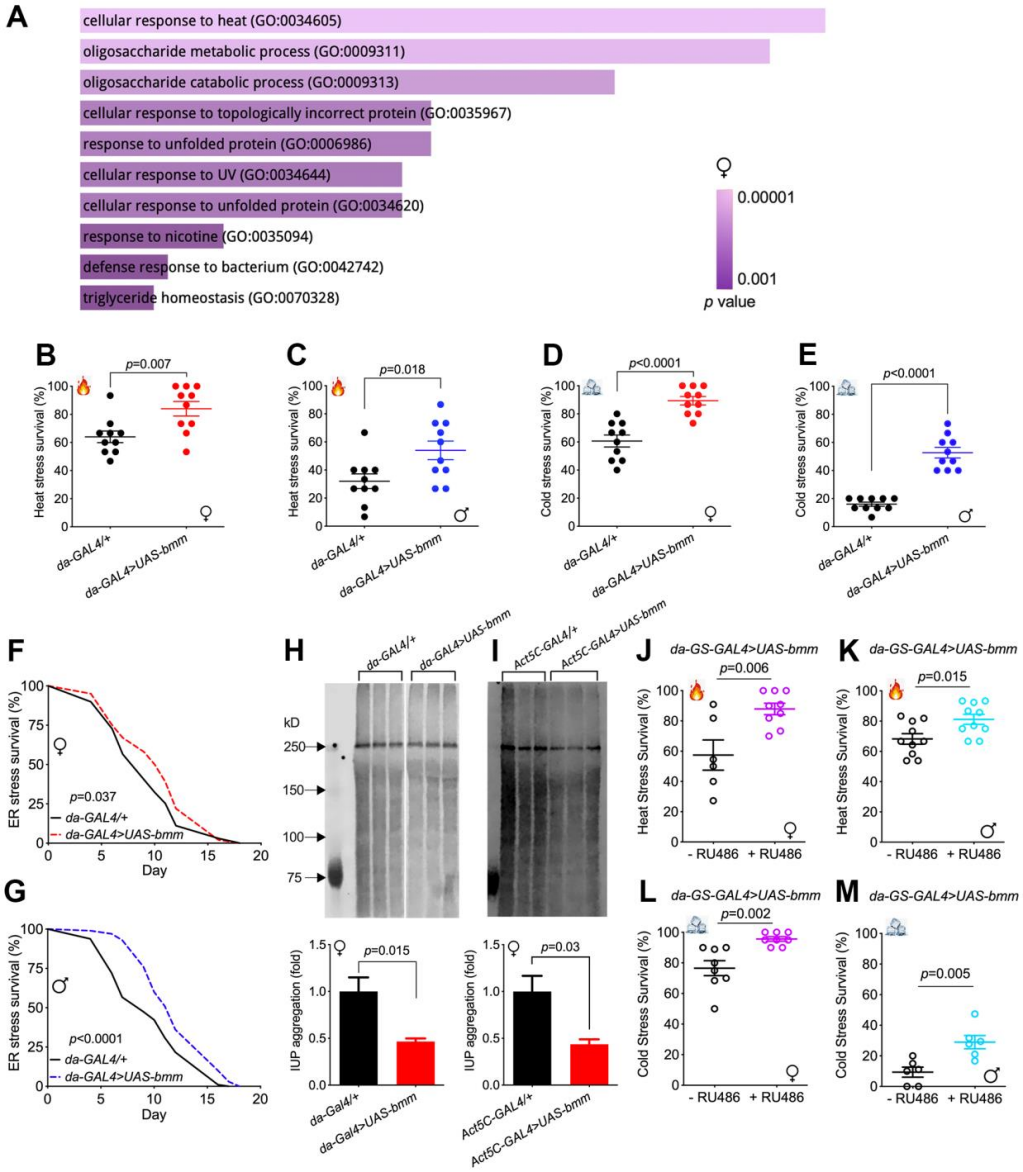
***bmm* overexpression enhances stress resistance and improves protein homeostasis.** (**A**) Gene ontology enrichment analysis using FlyEnrichr identified the top 10 significantly enriched biological processes for the total DEGs identified in RNA-seq analysis for female *da-GAL4/+* vs. *da-GAL4>UAS-bmm* flies, ranked by *p* value. The length of the bar represents the significance of the corresponding specific biological process. (**B**, **C**) Heat stress resistance test in *da-GAL4/+* vs. *da-GAL4>UAS-bmm* flies. (**D**, **E**) Cold stress tolerance test in *da-GAL4/+* vs. *da-GAL4>UAS-bmm* flies. Data are shown as mean±SEM and analyzed by two-tailed Student *t*-test in (**B**–**E**). n=150 for each group. (**F**, **G**) The survival curves upon tunicamycin-induced ER stress of *da-GAL4/+* vs. *da-GAL4>UAS-bmm* flies*.* n=97-100 for each group and *p* value was determined by log-rank test. (**H**, **I**) Insoluble ubiquitinated protein (IUP) aggregation measurements by Western blotting in old female control vs. *bmm* overexpression flies at 35 to 40 days of age. n=3-6 replicates for each group, and each replicate protein was extracted from 10 flies. Samples in (**H**) were run on the same blot with the middle lanes of unrelated treatment groups cropped out. Data are shown as mean±SEM and statistical analysis was carried out by two-tailed Student *t*-test. (**J**, **K**) Heat stress resistance test in inducible *da-GS-GAL4>UAS-bmm* flies with or without RU486 induction. n=64-139 for each group. (**L**, **M**) Cold stress tolerance test in inducible *da-GS-GAL4>UAS-bmm* flies with or without RU486 induction. n=104-160 for each group. Data are shown as mean±SEM and analyzed by two-tailed Student *t*-test in (**H**–**M**). See also Supplementary Figures 3, 4.

To understand the effect of *bmm* overexpression more comprehensively, we performed RNA-seq analysis of whole flies. We identified 148 differentially expressed genes in females and 205 differentially expressed genes in males in response to *bmm* overexpression (FDR < 0.05 and absolute value (log_2_(fold-change)) > 1) (Supplementary Figure 3A, 3B). Pathway enrichment analysis of the differentiated genes revealed that cellular responses to various forms of stress were upregulated in female *bmm* overexpression flies (Figure 3A). Among the significantly differentially expressed genes, heat shock protein HSP70 family members including *Hsp70Ba, Hsp70Bb* were highly upregulated in both female and male *bmm* overexpression flies (Supplementary Figure 3C–3F).

Based on the changes in expression of heat shock proteins, we subjected flies to heat stress by incubating the flies at 37° C. Female and male flies overexpressing *bmm* showed robust resistance to heat stress, with significantly higher survival rates than the control flies (Figure 3B, 3C and Supplementary Figure 4A), with the exception that male *bmm* overexpression flies did not show better survival when compared with *+/UAS-bmm* controls (Supplementary Figure 4B). Up-regulation of *Hsp70s* has also been reported to be important for cold tolerance [18]. When subjected to cold challenge, *bmm* overexpression also enabled stronger resistance against cold stress in both female and male flies (Figure 3D, 3E and Supplementary Figure 4C, 4D). Since Hsp70 proteins are molecular chaperones that play pivotal roles in protein folding and maintaining proteostasis [19], we subjected flies to tunicamycin-induced endoplasmic reticulum (ER) stress. Both female and male *bmm* overexpression flies demonstrated higher survival under ER stress conditions (Figure 3F, 3G and Supplementary Figure 4E, 4F). In addition, insoluble ubiquitinated protein (IUP) aggregation, which is a hallmark of aging [20, 21], was significantly decreased in *da-GAL4>UAS-bmm* and *Act5C-GAL4>UAS-bmm* flies, but this effect was limited to females (Figure 3H, 3I and Supplementary Figure 4G–4L). Increased resistance to both heat and cold stress was also observed in response to RU486-mediated induction in adult female and male *da-GS-GAL4>UAS-bmm* flies (Figure 3J–3M), but not in control *da-GS-GAL4/+* flies (Supplementary Figure 4M–4P), suggesting that the beneficial effects of *bmm* are not due to changes in development. Taken together, these data show that *bmm* overexpression increases the resistance to various stress conditions and preserves protein homeostasis in aged flies.

### *bmm* overexpression increases mitochondrial biogenesis and oxidative metabolism

**Figure 4:**
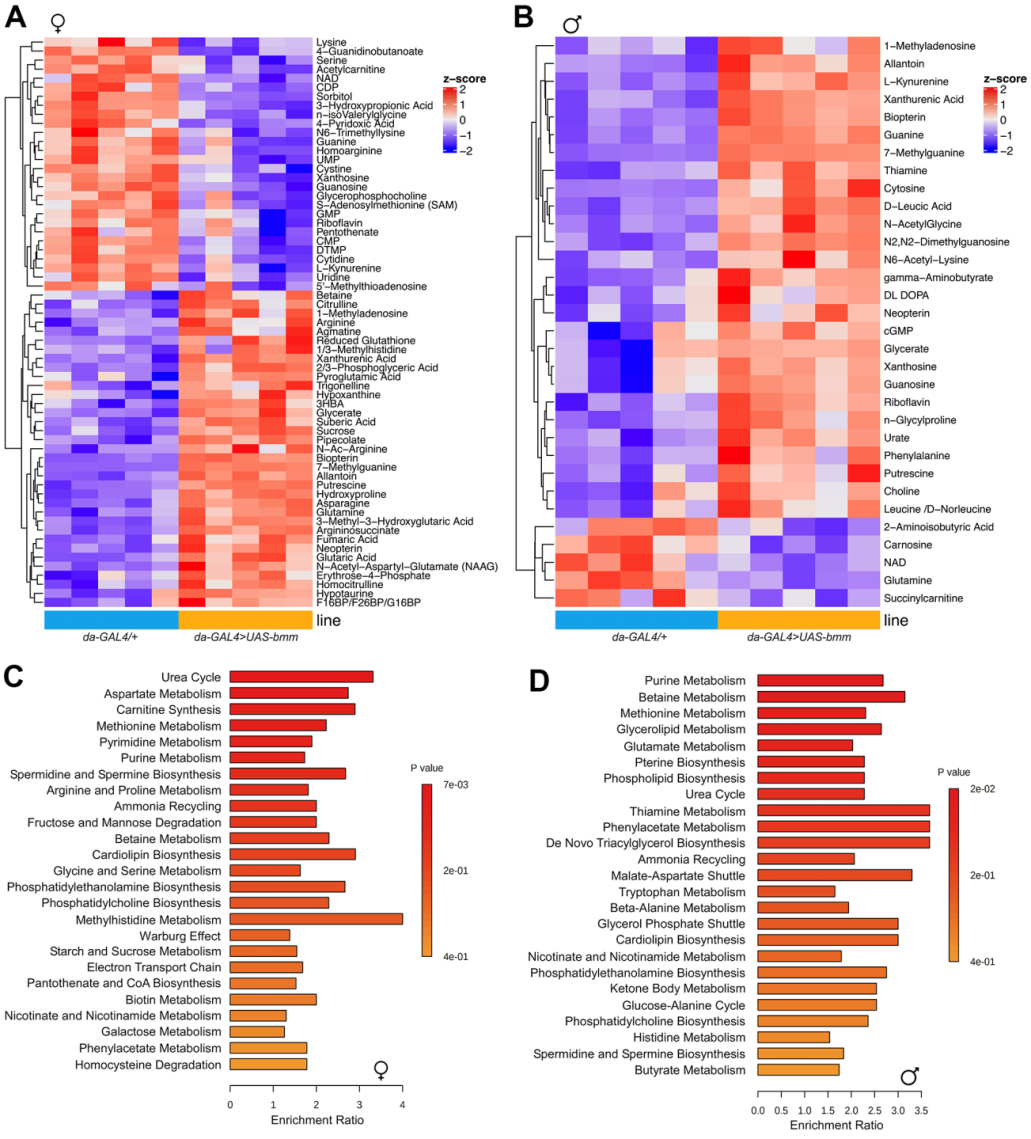
***bmm* overexpression increases mitochondrial biogenesis and oxidative metabolism.** (**A**) Heatmap of 63 significantly differentiated metabolites identified between *da-GAL4/+* and *da-GAL4>UAS-bmm* female flies by targeted metabolomics analysis. (**B**) Heatmap of 32 significantly differentiated metabolites identified between *da-GAL4/+* and *da-GAL4>UAS-bmm* male flies by targeted metabolomics analysis. (**C**, **D**) Pathway analysis of significantly differentiated metabolites identified between *da-GAL4/+* and *da-GAL4>UAS-bmm* flies. Each row represents a pathway and each bar indicates the enrichment of the metabolites detected in a given pathway. Color bar represents significance. See also Supplementary Figure 5.

**Figure 5:**
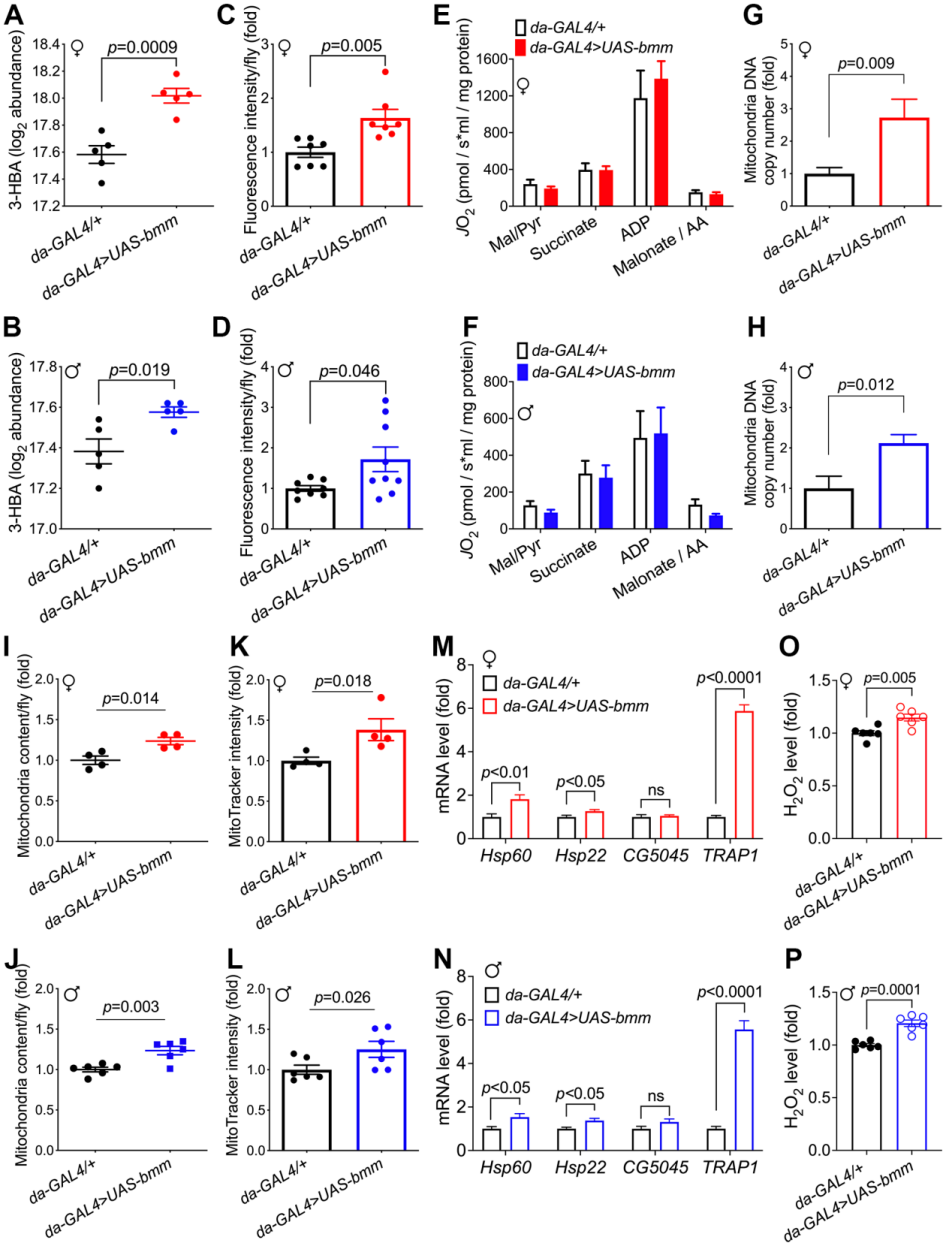
***bmm* overexpression increases mitochondrial biogenesis and oxidative metabolism.** (**A**, **B**) Level of 3-hydroxybutyrate (log_2_ abundance) in *da-GAL4/+* vs. *da-GAL4>UAS-bmm* flies. n=5 replicates, and each replicate contained 20 flies. FDR=0.0008 for *bmm* overexpression vs. control females, and FDR=0.13 for males. (**C**, **D**) β-oxidation rate measurement using fatty acid oxidation direct detection reagent, FAOblue (10 μM for 1 hour incubation). Each group consists of n=7-9 replicates, and each replicate sample was extracted from 20 flies. Data are shown as mean±SEM. Statistical analysis was carried out by two-tailed Student *t*-test in (**A**–**D**). (**E**, **F**) Oxygen consumption rate measurements of isolated mitochondria from *da-GAL4/+* and *da-GAL4>UAS-bmm* flies, when stimulated by indicated reagents. Data are shown as mean±SEM and normalized by mitochondria protein. Statistical analysis was carried out by two-way ANOVA. n=5-6 replicates, and each replicate contained mitochondria extracted from 50 flies. Mal/Pyr: malate + Pyruvate; AA: antimycin A. (**G**, **H**) Quantification of mitochondrial DNA copy number in *da-GAL4/+* vs. *da-GAL4>UAS-bmm* flies. Data are shown as fold change of mitochondrial *Cytb* DNA normalized to nuclear histone DNA. n=6-11 replicates, and each replicate was extracted from 10 flies. (**I**, **J**) Quantification of mitochondrial content in *da-GAL4/+* vs. *da-GAL4>UAS-bmm* flies. n=4 replicates for females, and n=6 replicates for males. Mitochondria were isolated from 50 flies for each replicate, and protein content was quantified by BCA analysis. (**K**, **L**) MitoTracker intensity measurement of *da-GAL4/+* vs. *da-GAL4>UAS-bmm* flies to assess mitochondrial mass. n=4 replicates for females, and n=6 replicates for males with 50 flies for each replicate. (**M**, **N**) Mitochondrial UPR genes mRNA expression level measurement in *da-GAL4/+* vs. *da-GAL4>UAS-bmm* flies. n=7-11 replicates. (**O**, **P**) H_2_O_2_ level measurement in *da-GAL4/+* vs. *da-GAL4>UAS-bmm* flies. n=6 replicates. Data are shown as mean±SEM. Statistical analysis was carried out by two-tailed Student *t*-test. See also Supplementary Figure 6.

To further investigate the metabolic changes underlying the physiological effects of *bmm* overexpression, we performed targeted metabolomics analysis on whole flies [22]. In total, 202 metabolites were detected, among which 63 metabolites were significantly different between *bmm* overexpression and control female flies (Figure 4A), and 32 significant differential metabolites in male flies (Figure 4B). Pathway analysis revealed that *bmm* overexpression impacted amino acid metabolism, the urea cycle, and purine/pyrimidine metabolism among others (Figure 4C, 4D and Supplementary Figure 5A, 5B). In addition, enriched pathways also included cardiolipin biosynthesis and electron transport chain (Figure 4C, 4D), suggesting alterations in mitochondrial biogenesis and metabolism. Indeed, and in line with previous studies showing lipolysis promotes increased β-oxidation [23], *bmm* overexpression flies had increased levels of ketone body 3-Hydroxybutyric acid (3-HBA) (Figure 5A, 5B) and increased rates of fatty acid oxidation compared with controls (Figure 5C, 5D). Yet, we did not observe significant differences of oxygen consumption rate from mitochondria isolated from control and *bmm* overexpression flies when normalized by mitochondrial protein (Figure 5E, 5F). However, mitochondrial DNA copy number was nearly doubled in both female and male *bmm* overexpression flies compared with controls (Figure 5G, 5H), which suggested the increase in fatty acid oxidation in *da-GAL4>UAS-bmm* flies was due to increased mitochondrial biogenesis triggered by *bmm* overexpression rather than increased mitochondrial function. This was further confirmed by mitochondrial protein concentration measurements (Figure 5I, 5J and Supplementary Figure 6A, 6B) as well as MitoTracker staining in *bmm* overexpression flies (Figure 5K, 5L). These results are consistent with previous studies in mammalian cells linking ATGL with increased mitochondrial biogenesis [24]. With mitoDNA content increased, there may be an imbalance between mitochondria and nucleus encoded mitochondrial proteins, leading to mitochondrial unfolded protein response (UPR^mt^) [25]. Marginal yet significant increases in expression was detected in the UPR^mt^ genes *Hsp60* and *Hsp22,* and a ~5 fold increase of mRNA expression level of *TRAP1* gene was observed in both female and male *bmm* overexpression flies (Figure 5M, 5N), suggesting an induction of the UPR^mt^ as a result of increased *bmm*. Collectively, these data suggest that increased mitochondrial content and dynamics may contribute to the observed phenotypes.

Among the significantly changed metabolites, reduced glutathione (GSH), a major antioxidant, was observed to be increased in female *bmm* overexpression flies (Supplementary Figure 6C), but this was not the case for the male flies (Supplementary Figure 6D). A recent study reports that sustained β-oxidation via ATGL-1 activation in *C. elegans* evokes mitochondrial stress response and triggers a feedback transcriptional loop shielding the organism from life-shortening mitochondrial stress in the face of continuous fat oxidation [26]. Consistent with the hypothesis that *bmm* overexpression flies would be subjected to higher oxidative pressure, significantly higher ROS level was observed in both female and male *bmm* overexpression flies (Figure 5O, 5P), which may also contribute to the UPR^mt^ induction [25]. In addition, female *bmm* overexpression flies were more sensitive to increased oxidative stress conditions when challenged by both high and low concentrations of H_2_O_2_ (Supplementary Figure 6E–6H). These data suggest that *bmm* overexpression female flies maintain a delicate balance in managing oxidative stress such that antioxidant defenses are kept compatible with the elevated rates of fatty acid oxidation to ensure a robust energy supply.

### *bmm* overexpression has marginal or no effects on lifespan in *Drosophila*

**Figure 6:**
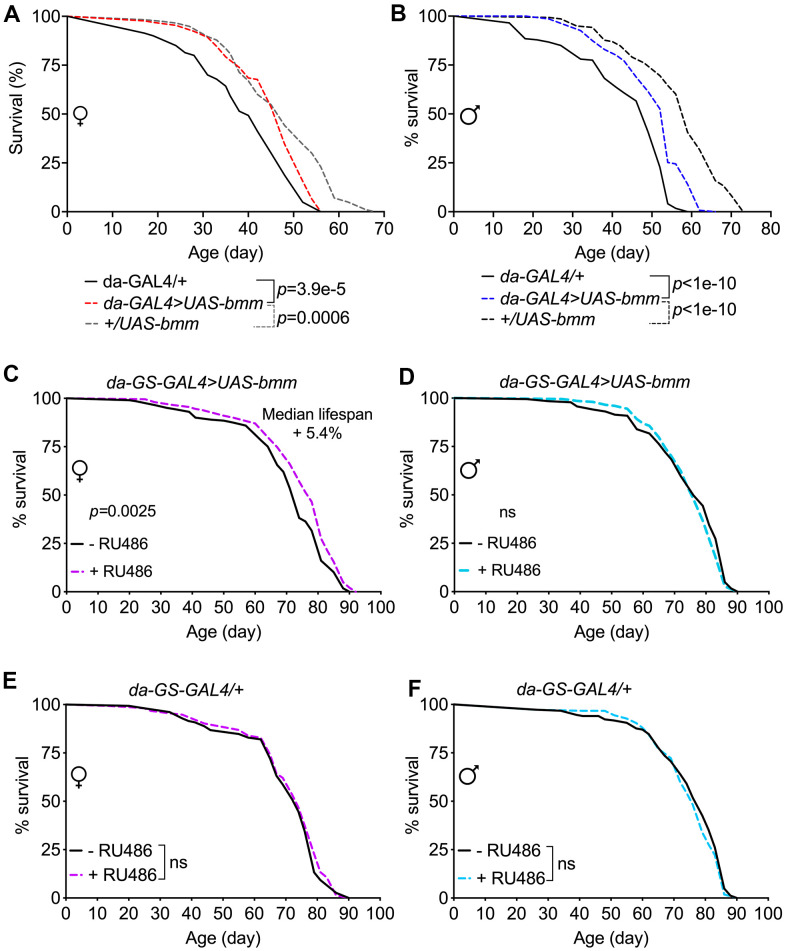
***bmm* overexpression has marginal effects on lifespan in *Drosophila*.** (**A**, **B**) Kaplan-Meier curves of *da-GAL4>UAS-bmm* vs. control *da-GAL4/+* and *+/UAS-bmm* flies. (**C**, **D**) Kaplan-Meier curves of inducible *da-GS-GAL4>UAS-bmm* with or without 50 μM RU486 induction*.* (**E**, **F**) Kaplan-Meier curves of inducible *da-GS-GAL4/+* control flies with or without 50 μM RU486 induction*.* n for each group and *p*-value by log-rank analysis are listed in Supplementary Table 1.

Given the robust effects of *bmm* overexpression on numerous physiological parameters, we investigated if these effects translated to alterations in lifespan. Although flies overexpressing *bmm* using the *da*-*GAL4* driver had significantly prolonged median lifespan when compared with *da-GAL4/+* flies, they were much shorter-lived when compared with *+/UAS-bmm* control flies (Figure 6A, 6B and Supplementary Table 1). The longer lifespan of *+/UAS-bmm* control flies than the *GAL4/+* control flies (Figure 6A, 6B) was consistent with previously reported lifespan phenotypes [27], which suggests potentially adverse health effects imposed by the excess exogenous expression of GAL4 protein especially in the later life stage. Furthermore, using inducible *da-GS-GAL4* line, we observed only marginal lifespan extension effects in female flies with *bmm* overexpression upon RU486 induction (5.4% median lifespan increase) (Figure 6C), and no lifespan extension effects were observed in male flies (Figure 6D); control *da-GS-GAL4/+* flies showed no lifespan changes under vehicle and RU486 induction conditions (Figure 6E, 6F). Thus, we conclude that increased expression of *bmm* results in marginal or no lifespan extension.

## DISCUSSION

**Figure 7:**
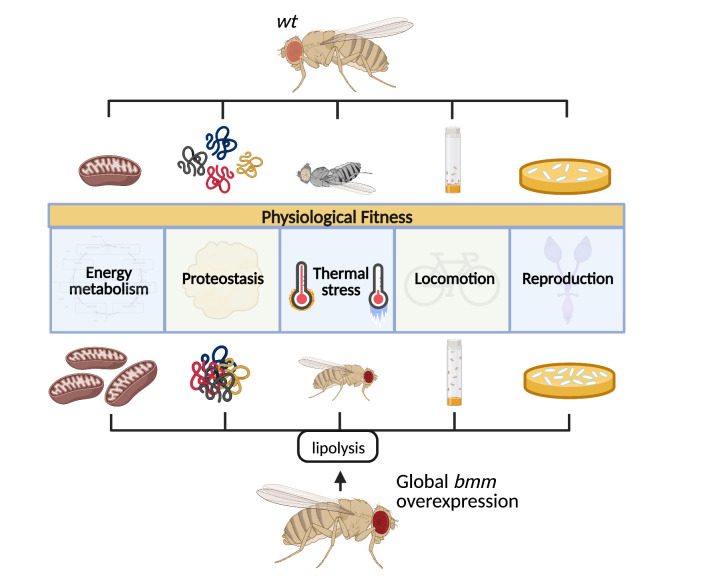
Increased systemic lipolysis through overexpression of the lipase *bmm* robustly promotes various health markers including increased fecundity, sustained locomotion capacity, enhanced stress resistance, and maintained proteostasis in flies.

Lipid metabolism is increasingly recognized to be an important regulator of aging and health. Herein, we show that overexpression of the cytosolic lipase *bmm,* which increased lipid catabolism*,* promoted numerous physiological parameters including fecundity, locomotion, oxidative metabolism, proteostasis, and resistance to thermal and ER stress (Figure 7). Collectively, these studies highlight lipid catabolism as a key metabolic and signaling node that has robust and wide-ranging benefits on physiological fitness. While outcomes in males and females in response to *bmm* overexpression were generally changed in the same direction, sex differences were often observed regarding the magnitude of change. These differences were expected as *bmm* is more highly expressed in males, which are leaner, and ablation of *bmm* attenuates differences in TAG storage and turnover between males and females [16].

One hallmark of aging is the accumulation of abnormal proteins, which causes protein toxicity and subsequent maladaptive stress responses [28]. Our data showed that *bmm* overexpression increased stress responses to suboptimal heat and cold conditions as well as ER stress. Heat shock proteins (Hsps) regulate both stress resistance and aging by facilitating protein refolding and turnover [19, 28]. Hsp70 family members possess chaperone-like functions and are tightly connected with cellular stress responses. Their induction is closely related to tolerance to high temperature and confers stress resistance in *Drosophila* larvae [29]. Our study showed substantial induction of *Hsp70* expression in *bmm* overexpression flies. As animals age, dysfunction of the protein quality control machineries and accumulation of abnormal protein aggregates occur [28], which induces gene expression of *Hsps* through the transcription factor HSF [19]. These studies provide important clues for further investigation on the potential mechanisms underlying the transcriptional induction of *Hsp70* gene expression driven by *bmm* overexpression. Polymorphisms of the three *HSP70* genes, *HSPA1A, HSPA1B* and *HSPA1L* have been found to be significantly associated with human longevity and survival [30]. Furthermore, lymphoblasts from human centenarians maintain the transcriptional response of the *Hsp70* gene to stress similar to young subjects [31]. In *Drosophila*, heat-induced expression of *Hsp70* increases lifespan at normal temperatures [32] although these findings have been challenged [33]. Collectively, this work identifies lipolysis as an upstream regulator of stress response via Hsp70 induction, a key phenotypical outcome of the current studies.

Our lipidomic studies revealed that *bmm* overexpression had robust TAG lowering effects, which were expected. In addition, we observed significantly decreased fatty acids in females and with more limited and species-specific reductions in males. A recent study has found that an isocaloric moderately high-fat diet extends lifespan in both male rats and *Drosophila* by decreasing fatty acids through increasing rates of their catabolism [34]. Given that *bmm* overexpression robustly increased mitochondria abundance and oxidative capacity, we postulate that the observed reductions in fatty acids resulted from enhanced utilization. As suggested previously, these changes are likely key metabolic adaptations that allow for increased disposal to match the elevated fatty acid supply resulting from higher rates of lipolysis [23, 24]. In addition, the metabolomic analysis also highlighted many conserved changes between males and females flies with *bmm* overexpression. However, several metabolites involved in purine metabolism, one of the top pathways altered in response to *bmm* overexpression, were reciprocally regulated. Females had reduced amounts of the purine pathway metabolites guanine, xanthosine, and guanosine and increased glutamine with *bmm* overexpression, whereas these metabolites were changed reciprocally in males. Since *bmm* drives fatty acid oxidation, these data implicate differential partitioning of glucose into the pentose phosphate pathway and purine biosynthesis between males and females. However, the underlying mechanisms through which *bmm* elicits these sex-specific effects remains to be determined.

Along with increased mitochondria content, mitochondrial unfolded protein response was induced in *bmm* overexpression flies. Robust increased expression of *TRAP1* gene, which encodes a mitochondrial chaperone protein of the heat shock protein (HSP90) family, was detected in both female and male *bmm* overexpression flies. Previous study has found that the overexpression of TRAP1 in *Drosophila* extends healthspan including enhanced stress resistance, locomotor activity and fertility [27]. UPR^mt^ induces epigenetic changes to promote longevity was reported in *C. elegans* [35]. A feedback loop involving the mito-nuclear through transcription factor ATFS-1, which balances the UPR^mt^ and lipid metabolism was also reported in *C. elegans* [26]*.* In addition, cross-communication between mitochondrial-to-cytosolic heat shock proteins has been discovered to be involved in fatty acid metabolism for ensuring cellular homeostasis [36]. It is of our great interest to further investigate the coordinated cross-compartments stress regulation, given the induction of UPR^mt^ and the robust increase of *Hsp70s* expression observed in *bmm* overexpression flies in our study. Taken together, these data suggest that lipolysis plays a key regulatory role in stress responses.

In our model, *bmm* overexpression female flies had robustly increased fecundity. Uncoupling of longevity and fecundity has been reported in other conditions [37, 38], suggesting the costs of reproduction might not necessarily be due to competitive trade-offs of resources for other physiological fitness such as locomotion and stress resistance observed in the *bmm* overexpression flies. In addition, the increased food intake in female flies may provide surplus resources, which alternatively explains the increased fecundity we observed in *bmm* overexpression female flies.

Collectively, these studies reveal diverse beneficial effects of global elevation of lipolysis on physiological fitness. This work provides additional rationale for pursuing therapeutic approaches, as done previously [39], that enhance lipolysis to mitigate metabolic and aging-related diseases.

## MATERIALS AND METHODS

### Fly strains and husbandry

The *UAS-bmm* line and its original background line *w^1118^* were obtained from Dr. Ronald P. Kühnlein. *daughterless-GAL4* line was received from Dr. Aidan Peterson, and *daughterless-GeneSwitch-GAL4* line was a gift from Dr. David W. Walker. *Act5C-GAL4* line was obtained from the Bloomington Stock Center. Upon arrival, the *UAS-bmm, daughterless-GAL4,* and *Act5C-GAL4* lines were backcrossed to *w^1118^* control strain for more than 10 generations before subjected to studies. Fly stocks maintenance and larval growth used Bloomington *Drosophila* Stock Center standard cornmeal medium (https://bdsc.indiana.edu/information/recipes/bloomfood.html). Adult flies for all experiments were carried out using standard SY medium [40, 41]: 10% w/v sucrose (Genesee Scientific, Cat#: 62-112), 10% w/v yeast (Genesee Scientific, Cat#: 62-106), 1.5% w/v agar (Mooragar Inc.), supplemented with Tegosept as preservative (Genesee Scientific, Cat#: 20-259, 30 mL/L of 10% w/v Tegosept in 95% EtOH added once the food had cooled below 65° C). RU486 (TCI America, Cat#: M1732) was dissolved in 80% ethanol and mixed thoroughly into food cooled below 65° C at a final concentration of 50 μM; the same volume of 80% ethanol was added as vehicle control.

### Lifespan assay

Lifespan studies were conducted based on previously published procedures [41]. Flies were reared at a standard density (~ 250-300 eggs per 200 mL bottle) using synchronized eggs laid within 24 hours. Upon emerging, flies were transferred onto fresh SY medium and allowed 48 hours to mature and mate before sorted into single sexes under light CO_2_ anesthesia at 15-20 flies per vial, with 10 vials per condition for most lifespan studies. Flies were transferred to new vials with fresh food three times a week, at which point the death events and censored flies were recorded. All lifespan studies were conducted at 25° C, 65% relative humidity in a 12 h light: 12 h dark cycle. Unless otherwise noted, each lifespan study was repeated at least twice. Statistical analysis of survival curves was evaluated by log-rank test; *p* values and details of fly numbers used in studies are provided in the Figures and Supplementary Table 1.

### Fecundity assay

For egg-laying assays, 5-10 once-mated female flies per vial were transferred into a new vial with fresh food and allowed to lay eggs for 24 hours, and the number of eggs laid per vial was counted. Data were calculated as the number of eggs laid per female fly per 24 hour. Typically, 10 biological replicates per condition were conducted for each assay. For assessing pupae production by aged flies, 28-day-old female flies or 30-day-old male flies were paired with 2-day-old *w^1118^* flies of the opposite sex (female *w^1118^* flies were virgins). All flies were discarded after 48 hours. The vials were kept for 7 days at 25° C, then pupae number was counted. Data were calculated as the number of pupae produced per fly.

### Stress and starvation assays

Flies were sorted and maintained on SY medium as in lifespan experiments described previously. Flies aged 1-2 weeks were used for stress studies. For heat stress studies, flies were incubated in a 37° C water bath for 2 to 3 hours in vials completely immersed in water up to the height of the cotton plug. The number of dead flies were counted after 24 hours recovery. For cold stress studies, fly vials were similarly immersed in icy water for 16 hours, and the number of dead flies were counted after 24 hours recovery. Tunicamycin-induced ER stress, flies were transferred into vials containing filter paper soaked with 10% w/v sucrose solution with 20 μM tunicamycin (Tocris Bioscience, Cat#: 3516). Flies were transferred into new vials with fresh reagent every other day, and death events were counted every day throughout the assay. For H_2_O_2_ stress studies, flies were transferred into vials containing filter paper soaked with 10% w/v sucrose solution with 3% or 1% H_2_O_2_ (Sigma-Aldrich, Cat#: H1009). Flies were transferred into new vials with fresh reagent every other day, and the survival rates were calculated on day 5 and day 8, respectively. For starvation studies, flies were transferred and sustained on 1.5% w/v agar as a water source. Flies were transferred into new vials with fresh agar medium daily, and death events were counted every 2 hours during the day until all flies were dead. Unless otherwise noted, each stress experiment was repeated at least twice.

### Locomotion assay

Locomotion assays were carried out referring to the methodology described previously [42]. This climbing assay was used to score for negative geotaxis based on flies’ natural tendency to climb upwards against gravity. Flies were transferred into fresh vials without carbon dioxide anesthesia and allowed to acclimate to the environment without disturbance for 15-20 minutes. The vials were sharply tapped down on the surface of the bench to knock all flies down to the bottom of the vials, and simultaneously video-recorded for 10 seconds. During the 10 s window, flies climbing up reached a marked threshold after being tapped down to the bottom were counted as “climbers”. Detailed fly number and age at testing are indicated in the figure legends.

### Triglyceride assay

Triglyceride assays were carried out as previously described [43]. In brief, 7 female or 10 male flies per replicate were homogenized in PBS containing 0.05% Tween-20. Homogenates were heat-inactivated for 5 mins at 70° C and centrifuged for 3 mins at 3500 rpm. Samples were incubated with triglyceride reagent (Thermo Scientific #TR22421) at 37° C for 30 mins and read at 540 nm with BioTek Synergy™ Neo2 HTS Multi-Mode Plate Reader. Each group contained 6 replicates.

### Triglyceride hydrolase activity assay

At two weeks of age, 20 flies per biological replicate x 3 replicates per condition were homogenized in assay buffer [20 mM Tris-HCl (pH 8.0), 150 mM NaCl, 0.05% Triton X-100]. Samples were centrifuged at 15,000 x g at 4° C for 15 minutes. The infranatant was transferred and protein concentration was determined by BCA assay (Pierce, Cat#: 23225). 50 μg of protein samples was incubated with freshly prepared resorufin ester solution (0.3 mg/mL) (Sigma-Aldrich, Cat#: 424455) and read with a spectrophotometer every 10 min for 60 mins with excitation and emission wavelengths at 530 nm and 590 nm, respectively.

### RNA isolation, quantitative real-time PCR (qPCR) and RNA-seq analysis

Total RNAs were extracted from 10-20 flies per biological replicate using TRIzol reagent (Invitrogen, Cat#: 15596026) following the manufacturer protocol. RNAs were converted to cDNA using qScript cDNA synthesis kit (Quanta-bio, Cat#: 95047). Quantitative real-time PCR was performed using SYBR Green Master Mix (Applied Biosystems, Cat#: 4472918). Primers used in this study are as follows. Primers for *Hsp60, Hsp22,* and CG5045 genes are listed in previous publications [18, 27, 44].

*TRAP1*-forward: 5’-AGCAGCGTTCAATATCACCATT-3’

*TRAP1*-reverse: 5’-CTGCCTGGAACTCATGCTTGT-3’

*Rpl32*-forward: 5’-AGCATACAGGCCCAAGATCG-3’

*Rpl32*-reverse: 5’-TGTTGTCGATACCCTTGGGC-3’

High quality of RNAs passed QC were subjected to RNA-seq analysis. Unique dual-indexed (UDI) TruSeq stranded mRNA libraries were created and sequenced on NovaSeq 2x150-bp run with mean depth ≥ 20M reads, and mean quality scores were above Q30. RNA-seq data analysis was performed using the PURR-CHURP pipeline by the University of Minnesota Informatics Institute (UMII) at the University of Minnesota [45]. A subsample of 10,000 reads were sampled from each sample’s reads. This subsample of 10,000 reads was searched for contamination from ribosomal RNA using a representative rRNA sequence list from the SILVA database release 132. Trimmed reads were aligned to the reference *Drosophila* genome (version BDGP6.28) with HISAT2 [46]. Duplicate reads based on alignment position were marked with SAMTools. Processed alignments were filtered of reads that had a mapping quality of less than 60, and sorted by query name rather than position. Cleaned and name-sorted alignments were processed with the featureCounts tool from the subread package [47]. Raw per-gene counts were imported into R, and differential gene expression analysis was carried out using the edgeR package [48]. Genes were filtered by expression from the counts matrix before differential expression testing using the filtByExp() function. Exact tests were performed, and genes with false discovery rate (FDR) < 0.05 and absolute values (log_2_(fold-changes)) > 1 were considered significant. Volcano plots were generated using edgeR by *p*-values and log_2_ fold-changes with the EnhancedVolcano R package. Differential expression was performed by the Research Informatics Solutions group at the Minnesota Supercomputing Institute. Gene ontology enrichment analysis was performed using FlyEnrichr web server (https://maayanlab.cloud/FlyEnrichr/) [49, 50] with the input data of the sets of differentially expressed genes between control and *bmm* overexpression female flies, and the top 10 enriched biological processes ranked by *p* value were presented in the figure.

### Insoluble ubiquitinated protein (IUP) assay by Western blotting

Insoluble ubiquitinated protein (IUP) aggregation studies were performed as previously described [51]. 10 flies per replicate were homogenized in ice-cold PBS containing 1% Triton-X and protease inhibitors on ice. The homogenate was centrifuged at 14,000 x g for 10 mins at 4° C and the protein pellet was washed and resuspended in 50 mM Tris (pH7.5) and 2% SDS with protease inhibitors and sonicated. This SDS fraction containing the IUP was separated by SDS-PAGE using standard procedures. Primary Ubiquitin mouse monoclonal antibody (1:1000) (Cell signaling, Cat#: 3936), and secondary anti-mouse LI-COR antibody (1:5000) (Lincoln, NE, Cat#: 926-32212) were used with a LI-COR Odyssey Fc imaging system to detect fluorescent signaling, and the signal intensity quantification was performed using Image Studio Lite (LI-COR Biosciences).

### Mitochondria DNA copy number assay

DNA from 10 flies per biological replicate was isolated using Qiagen DNA isolation kit, per the manufacturer’s protocol. Mitochondria DNA copy number was determined by normalizing *Cytb* DNA to nuclear histone DNA using qPCR with SYBR green dye methods. The primers used in this study are as follows:

*Cytb*-forward: 5’-ACTCCTTTAGTAACACCTGCCC-3’

*Cytb*-reverse: 5’- TGGTCGAGCTCCAATTCAAGT -3’

*Histone*-forward: 5’- CACTCCTCGCCACTTACAGC-3’

*Histone*-reverse 5’- CCAGCGATGGTTGCCTTGA-3’

### Mitochondria isolation, mitochondria content evaluation, and Oroboros assay

Mitochondria were isolated as previously described [52]. 50 flies per biological replicate were gently crushed with pellet pestle (Fisher, Cat#: FS749520-0090) in ice-cold mitochondrial isolation medium (250 mM sucrose, 10 mM Tris-HCl (pH 7.4), 0.15 mM MgCl_2_) on ice. After being passed through a 100 μm filter and centrifuged at 1,000 x g for 5 mins at 4° C twice, the supernatant was centrifuged at 4,000 x g for 10 mins at 4° C, and the brownish-colored mitochondria pellet was washed and the mitochondrial protein concentration was determined by BCA assay (Pierce, Cat#: 23225). For MitoTracker studies, the isolated mitochondria were resuspended in the following buffer (105 mM k-MES, 30 mM KCL, 10 mM KH_2_PO_4_, 5 mM MgCl_2_-6H_2_O, 0.5 mg/ml fatty acid-free BSA, 1 mM EGTA (added freshly before usage), pH 7.2), and incubated with the fluorescent probe MitoTracker red (500 nM) (Invitrogen, Cat#: M22426) for 45 minutes, and the fluorescent signal was read at 665 nm with BioTek Synergy™ Neo2 HTS Multi-Mode Plate Reader. For Oroboros assay, the isolated mitochondria were subjected to measurement at 25° C using high-resolution respirometry (Oxygraph-2k, Oroboros) with the following sequential injections (final concentration in chamber): 5 mM pyruvate and 5 mM malate, 5 mM succinate, and 1 mM ADP to determine the maximal ADP stimulated respiration; followed with the addition of 1 μM malonate and 2.5 μM antimycin A to determine the residual nonmitochondrial oxygen consumption rate.

### Mitochondrial β-oxidation assay

β-oxidation rate measurement was carried out using fatty acid oxidation direct detection reagent, FAOblue (Funakoshi, Cat#: FDV-0033) at 10 μM for 1 hour [53]. The fluorescence intensity of coumarin dye released from FAOblue after complete β-oxidation was measured at excitation and emission wavelengths of 405 nm and 430-480 nm, respectively, with BioTek Synergy™ Neo2 HTS Multi-Mode Plate Reader. Each group consists of n=7-9 replicates, and each replicate sample was extracted from 20 flies.

### Mitochondrial ROS measurement

Mitochondrial ROS levels were evaluated by measuring cellular H_2_O_2_ content using Amplex Red Hydrogen Peroxide/Peroxidase Assay Kit (Invitrogen, Cat#: A22188) following the manufacturer’s protocol. Ten flies per replicate were homogenized in 1x reaction buffer. Samples were incubated with Amplex Red reagent/HRP working solution at room temperature for 30 mins and protected from light. Fluorescence signals were measured using excitation at 530 nm and emission at 590 nm with BioTek Synergy™ Neo2 HTS Multi-Mode Plate Reader. Each group contained 6 replicates.

### Feeding rate assay

Feeding rate was measured as previously described [54] using 5 females or 8 males per replicate, and 9 replicates per condition. Briefly, 0.05% w/v Erioglaucine disodium salt (FD&C blue No.1) (Sigma-Aldrich, Cat#: 861146) was thoroughly mixed into freshly prepared food after it had cooled off below 65° C. Adult flies were allowed to feed for 6 hours during the daytime, and then snap-frozen in liquid nitrogen. Flies were decapitated prior to analysis to avoid the eye pigment interference with the assay. The amount of blue dye in clarified fly homogenates was quantified spectrophotometrically for absorbance at 630 nm, reference at 675 nm with BioTek Synergy™ Neo2 HTS Multi-Mode Plate Reader.

### Metabolomic and lipidomic studies

Files were sorted and maintained as in the described lifespan studies. Upon reaching 30 days of age, 20 whole female or male flies per replicate, 5 replicates per condition, were collected by snap-freezing with liquid nitrogen. Metabolomic and lipidomic studies were carried out at the University of Washington (UW) Nathan Shock Center of Excellence in the Biology of Aging and the UW Northwest Metabolomics Research Center. For LC-MS metabolomics study, 365 molecules were targeted, from which 361 were metabolites and 4 were spiked stable isotope labeled internal standards (SILISs). Two sets of quality control (QC) samples - a lab QC (internal pooled human serum sample) and a sample QC (a pool of every 10 fly samples) - were included at the beginning and the end of each batch in order to monitor LC-MS assay performances as well as data reproducibility. Each metabolite was measured as peak area under MS curve. 202 metabolites and 4 SILISs were detected in at least one sample. The average and median coefficients of variation (CVs) in the sample quality control, QC(S), were 5.6% and 4.7%, respectively. For the lipidomic study, flies in each tube were homogenized, and an equal volume of homogenate was used for lipidomic extraction; BCA protein quantification was performed on the remaining sample. The lipidomic measurement was performed using a targeted mass spectrometry analysis designed to detect 1070 lipids, which are from 13 lipid classes (CE, CER, DAG, DCER, FFA, HCER, LCER, LPC, LPE, PC, PE, SM, TAG), and 568 lipids were detected. A lab QC (pooled human serum) and a sample QC (a pool of the fly samples) were included for every 10 samples at the beginning and the end of each batch. The median CV% was ~6% for the lab QCs and ~5% for the sample QCs.

### Metabolomic and lipidomic data analysis

Statistical analysis was carried out using R (version 4.0.0). We performed a median normalization for targeted metabolomics data where we adjusted the data, so all samples have the same median value of the metabolite abundance post log_2_ transformation. 192 metabolites with < 20% missingness and a CV < 20% in the pooled sample QC data were included in further analysis. After filtering, there were no missing values remaining; therefore, no imputation was performed. The targeted lipidomic data were in absolute concentration (in nmol/g of plasma (μM)); therefore, no further normalization was performed prior to statistical analysis. 349 lipids with < 20% missingness and a CV < 20% in the pooled sample QC data were included in further analysis. We used a quantile regression approach for the imputation of left-censored missing data (QRILC), which has been suggested as the favored imputation method for left-censored MNAR data [55]. This was implemented in the R imputeLCMD package. We fit linear models to the metabolomic data or the lipidomic data using the Bioconductor limma package [56] to assess the difference in abundance between experimental groups. The limma package uses empirical Bayes moderated statistics, which improves power by ‘borrowing strength’ between metabolites in order to moderate the residual variance [57]. We selected metabolites or lipids with a false discovery rate (FDR) of 5%. Comprehensive metabolite mapping and pathway analysis was performed using MetaboAnalystR package in R; based on MetaboAnalyst 5.0 (https://www.metaboanalyst.ca/home.xhtml) [58, 59].

### Method for heatmaps

In the heatmaps, z-scores were calculated for each row (each metabolite or lipid) and these were plotted instead of the normalized abundance values; this ensures that the abundance patterns/trends that we want to visualize are not overwhelmed by the abundance values. z-scores were computed by adjusting the data, by feature, to have a mean of zero and a standard deviation of 1. The heatmaps were generated using the ComplexHeatmap R package [60], where features were clustered via the hclust function with the ‘complete’ agglomeration method. Distance matrices for clustering were computed using ‘Euclidean’ distance.

### Statistics and reproducibility

GraphPad Prism 9.1 was used for generating graphs and performing the statistical analysis. For two groups comparison, unpaired, two-tailed Student *t*-test was used. For more than two groups comparison, one-way ANOVA with Tukey’s multiple comparisons test was performed. For two categorical variable comparisons, two-way ANOVA followed by Bonferroni multiple comparisons test was performed. For comparison of survival curves, log-rank test was carried out using PRISM and OASIS 2 [61]. *p* < 0.05 was considered as statistically significant. Data in figures are shown as mean±SEM. Detailed sample size for each experimental condition, statistical analysis method, median lifespan, and *p* value are listed in the graphs and Supplementary Table 1.

### Data availability statement

The RNA-seq data have been deposited to the Gene Expression Omnibus repository (GEO; https://www.ncbi.nlm.nih.gov/gds) with the accession number as GSE178816. Raw and processed gene expression values and annotation of the study groups used in this study are available at: https://www.ncbi.nlm.nih.gov/geo/query/acc.cgi?acc=GSE178816. Further information and requests for resources and reagents should be directed to and will be fulfilled by the corresponding author.

## Supplementary Material

Supplementary Figures

Supplementary Table 1
