# Supplementary material for: Systemic lipolysis promotes physiological fitness in Drosophila melanogaster
Source: Aging (Albany NY). 2022 Aug 30;14(16):6481–506. doi: 10.18632/aging.204251 (PMC9467406; doi:10.18632/aging.204251)
Supplement: Supplementary Figures [file aging-14-204251-s001.pdf]

SUPPLEMENTARY FIGURES

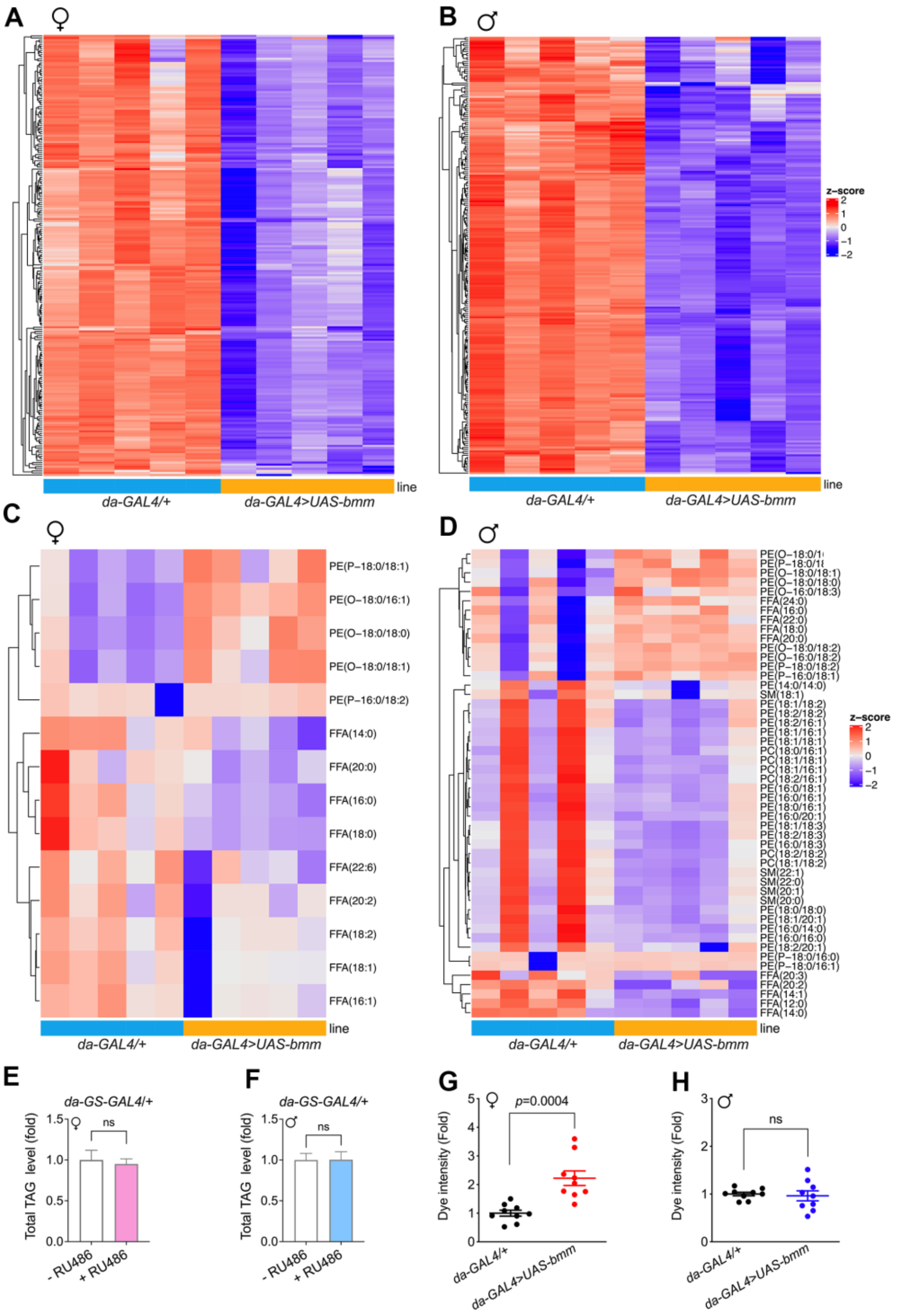

Supplementary Figure 1. Additional data related to global *bmm* overexpression effectively promotes lipolysis in *Drosophila*, related to Figure 1. (**A**, **B**) Heatmap of significantly different TAG lipids identified between *da-GAL4/+* and *da-GAL4>UAS-bmm* flies. (**C**, **D**) Heatmap of significantly different non-TAG lipid species identified between *da-GAL4/+* and *da-GAL4>UAS-bmm* flies. n=5 replicates, and each replicate contained 20 flies. (**E**, **F**) TAG content measurement in inducible *da-GS-GAL4/+* control flies with or without RU486 induction. n=6 replicates, and each replicate contained 7-10 flies. (**G**, **H**) Feeding rate was determined by adding brilliant blue dye into food and dye intensity was measured after 6 hours of feeding. n=9 replicates, and each replicate contained 5 flies for female group, and 8 flies for male group. Data are shown as mean±SEM. Statistical analysis was carried out by two-tailed Student *t*-test.

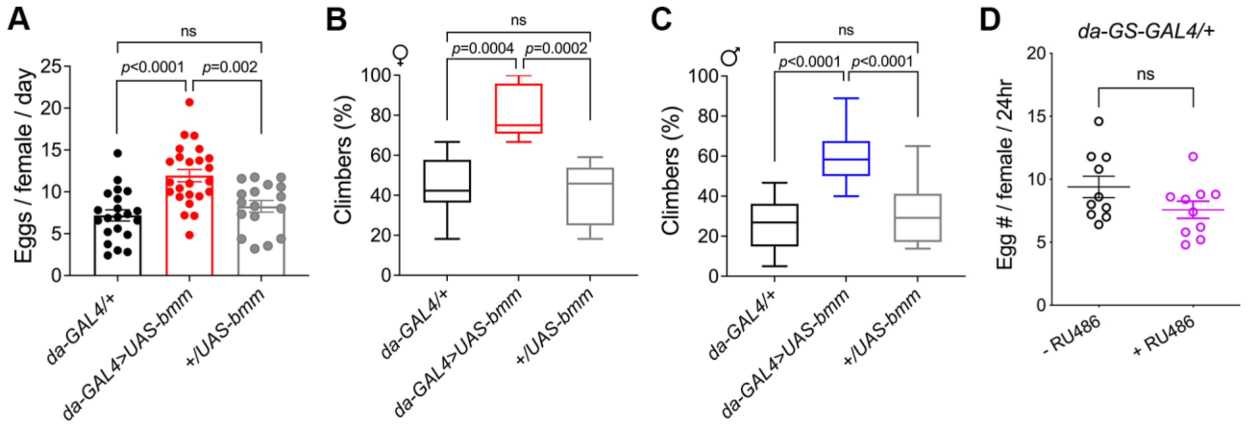

Supplementary Figure 2. Additional data related to *bmm* overexpression promotes physiological fitness in both female and male *Drosophila*, related to Figure 2. (**A**) Fecundity of 2-week-old *da-GAL4>UAS-bmm* vs. control *da-GAL4/+* and *+/UAS-bmm* flies. Data are shown as mean±SEM and analyzed by one-way ANOVA. (**B**, **C**) Locomotion analysis of *da-GAL4>UAS-bmm* vs. control *da-GAL4/+* and *+/UAS-bmm* flies at 30 days of age. Data are shown as box and whisker plot and analyzed by one-way ANOVA. (**D**) Fecundity of 2-week-old inducible *da-GS-GAL4/+* female flies with or without RU486 induction. n=50 for each group. Data are shown as mean±SEM and analyzed by two-tailed Student *t*-test.

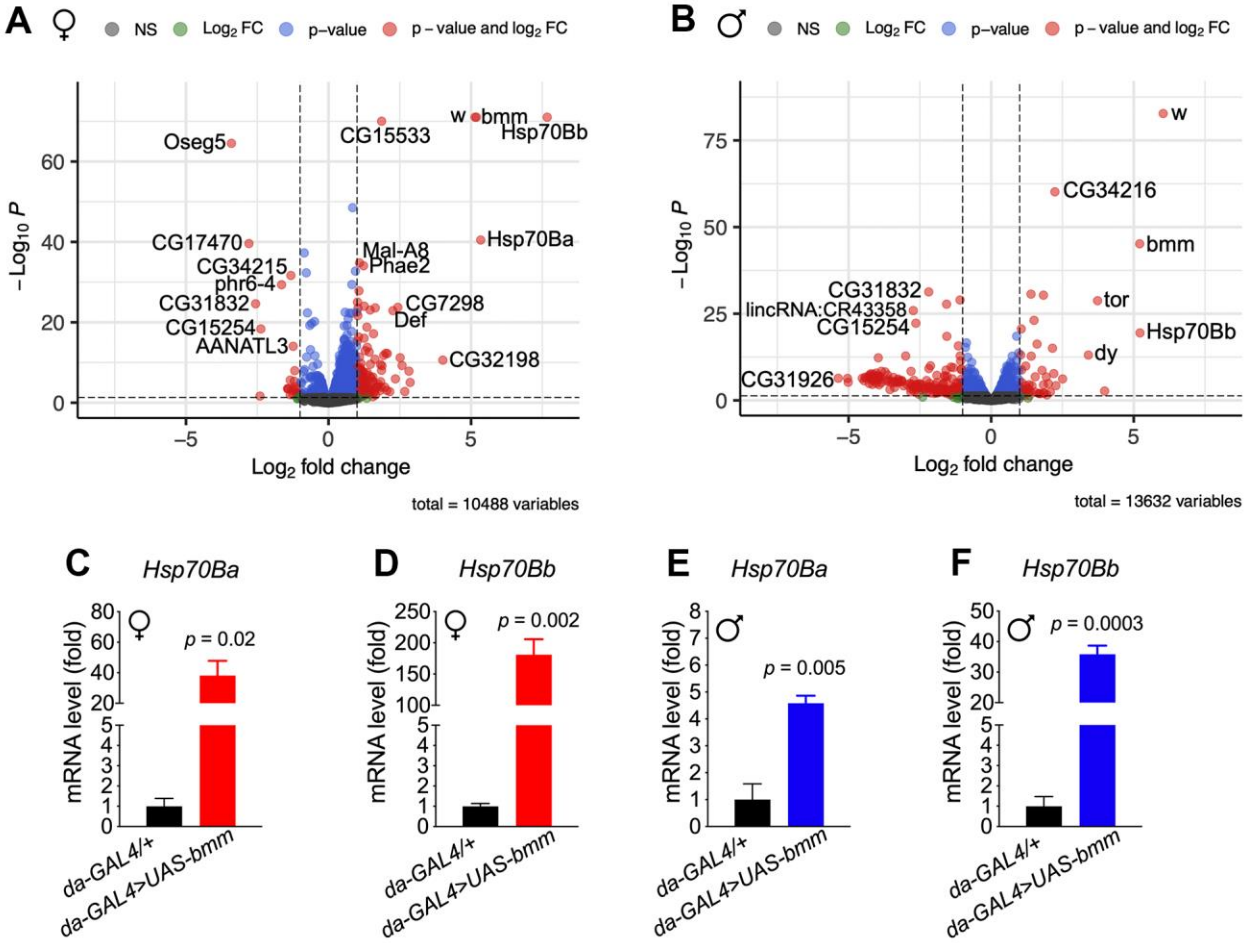

Supplementary Figure 3. Additional data related to *bmm* overexpression enhances stress resistance and improves protein homeostasis, related to Figure 3. (**A**, **B**) "Volcano plot" of genes by RNA-seq analysis with statistical significance against Log_2 fold change between *da-GAL4/+* and *da-GAL4>UAS-bmm* flies (p-value < 0.05, horizontal dotted line). Gene names for some of the most significantly differentially expressed genes (DEGs) are highlighted. NS=not significant. (**C**–**F**) *Hsp70Ba* and *Hsp70Bb* mRNA level measured by RNA-seq analysis in *da-GAL4/+* vs. *da-GAL4>UAS-bmm* flies. Data are shown as mean±SEM and analyzed by two-tailed Student t-test.

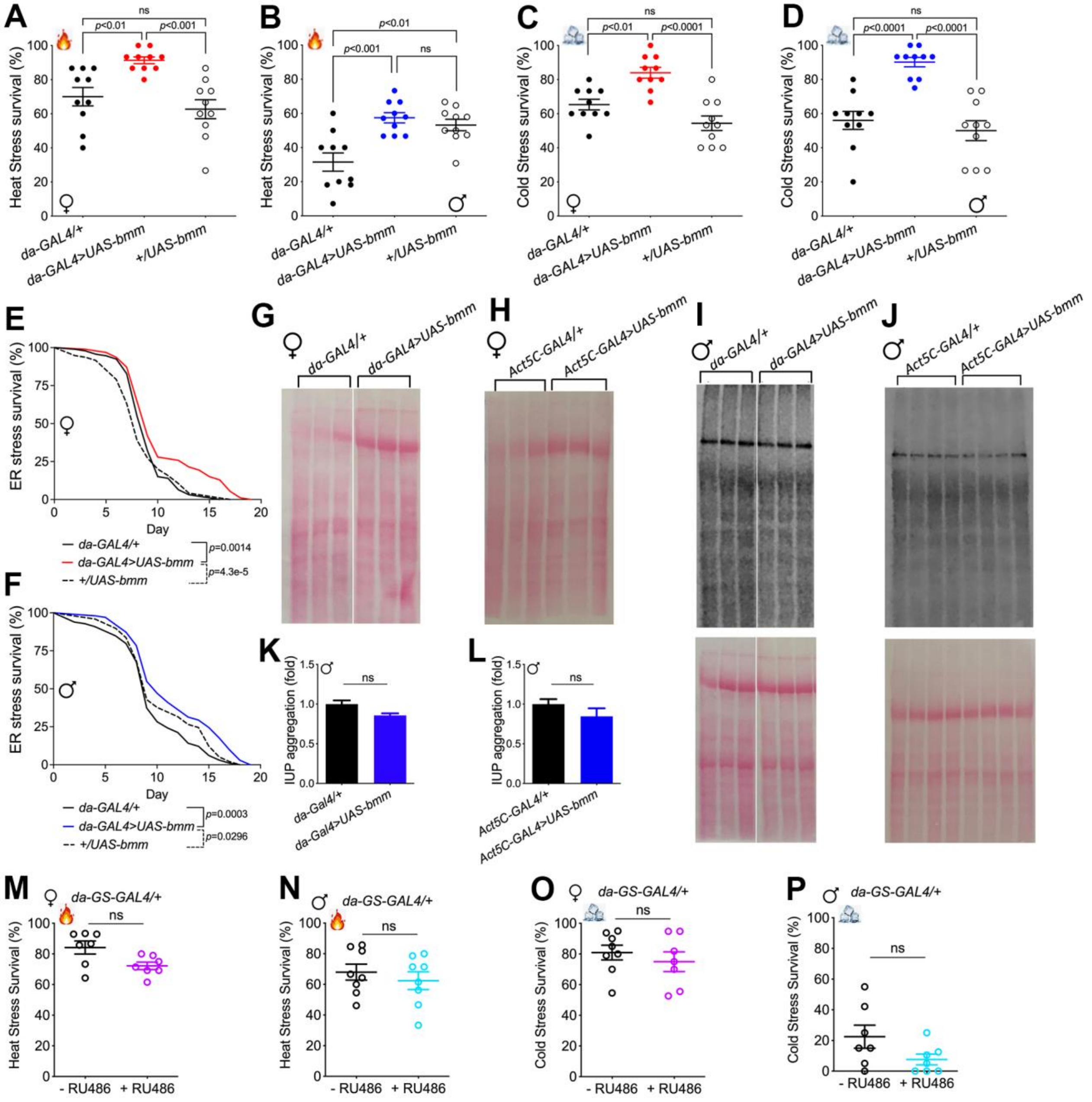

Supplementary Figure 4. Additional data related to *bmm* overexpression enhances stress resistance and improves protein homeostasis, related to Figure 3. (**A**, **B**) Heat stress resistance test in *da-GAL4>UAS-bmm* vs. control *da-GAL4/+* and *+/UAS-bmm* flies. (**C**, **D**) Cold stress tolerance test in *da-GAL4>UAS-bmm* vs. control *da-GAL4/+* and *+/UAS-bmm* flies. Data are shown as mean±SEM and analyzed by one-way ANOVA in (**A**–**D**). (**E**, **F**) The survival curves upon tunicamycin-induced ER stress in *da-GAL4>UAS-bmm* vs. control *da-GAL4/+* and *+/UAS-bmm* flies. n equaled about 100 flies for each group and *p* value was determined by log-rank test. (**G**, **H**) Ponceau S staining for normalization of insoluble ubiquitinated protein (IUP) aggregation intensity study in Figure 3H, 3I. n=3-6 replicates for each group, and each replicate protein was extracted from 10 flies. Samples in (**G**) were run on the same blot with the middle lanes of unrelated treatment groups cropped out. (**I**, **J**) IUP measurements by Western blotting and the corresponding Ponceau S staining in old male control vs. *bmm* overexpression flies at 35 to 40 days of age. n=3-4 replicates for each group, and each replicate protein was extracted from 10 flies. Samples in (**I**) were run on the same blot with the middle lanes of unrelated treatment groups cropped out. (**K**, **L**) IUP signal intensity quantification for (**I**, **J**) by Western blotting analysis. Ponceau S staining was served as loading control and was used for IUP signal quantification. (**M**, **N**) Heat stress resistance test in *da-GS-GAL4/+* control flies with or without RU486 induction. n=101-116 for each group. (**O**, **P**) Cold stress tolerance test in *da-GS-GAL4/+* control flies with or without RU486 induction. n=126-146 for each group. Data are shown as mean±SEM and analyzed by two-tailed Student *t*-test.

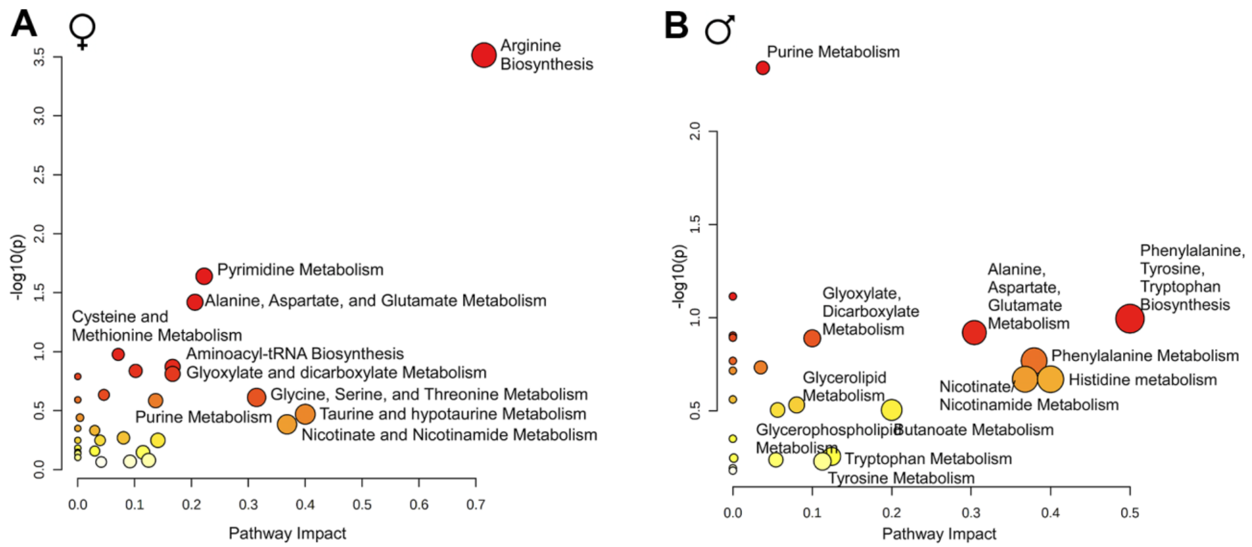

Supplementary Figure 5. Additional data related to *bmm* overexpression increases mitochondrial biogenesis and oxidative metabolism, related to Figure 4. (**A**, **B**) Enriched analysis of a metabolic pattern of interest in datasets generated by targeted metabolomics analysis on flies overexpressing *bmm*.

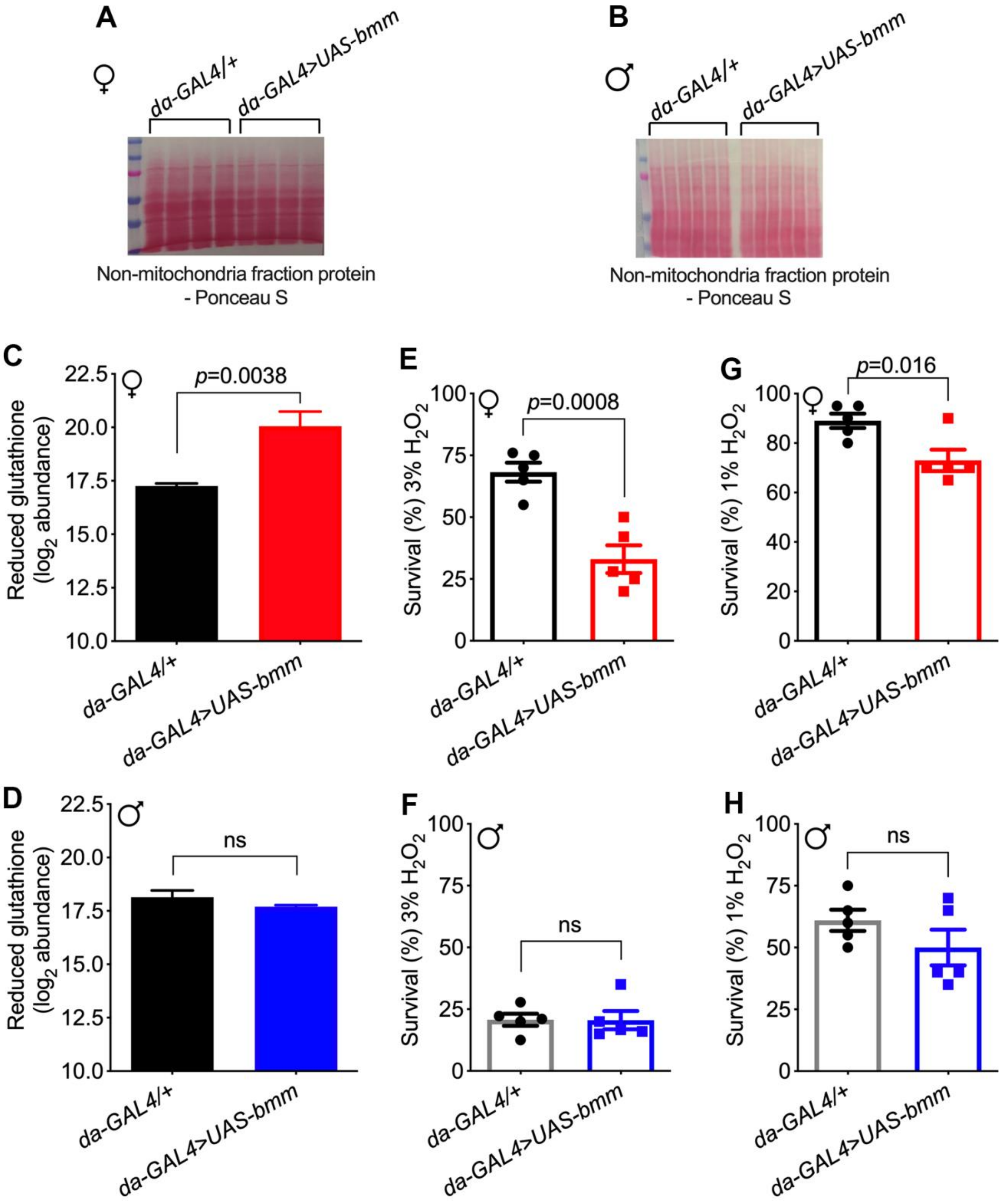

Supplementary Figure 6. Additional data related to *bmm* overexpression increases mitochondrial biogenesis and oxidative metabolism, related to Figure 5. (**A**, **B**) Ponceau S staining of non-mitochondria fraction protein corresponding to Figure 5I, 5J for mitochondria content quantification. n=4 replicates for female group, n=6 replicates for male group, and each replicate protein was extracted from 50 flies. (**C**, **D**) Level of reduced glutathione (log_2 abundance) in *da-GAL4/+* vs. *da-GAL4>UAS-bmm* flies. n=5 replicates, and each replicate contained 20 flies. The FDR=0.00026 for *bmm* overexpression vs. control female flies. (**E**, **F**) Survival rates of female and male flies under 3% H_2O_2 treatment. n=100 for each group. (**G**, **H**) Survival rates of female and male flies under 1% of H_2O_2 treatment. n=100 for each group. Data are shown as mean±SEM. Statistical analysis was carried out by two-tailed Student *t*-test in (**C**–**H**).
