# Supplementary material for: Systemic lipolysis promotes physiological fitness in Drosophila melanogaster
Source: Aging (Albany NY). 2022 Aug 30;14(16):6481–506. doi: 10.18632/aging.204251 (PMC9467406; doi:10.18632/aging.204251)
Supplement: Supplementary Table 1 [file aging-14-204251-s002.pdf]

SUPPLEMENTARY TABLE

Supplementary Table 1. *Drosophila* survival data, related to Figure 6.

Figure	Genotype	Sex	Replicate	Diet	n	Median lifespan (d)	Comparison	% change in Median lifespan	log-rank *p* value
Figure 6A	*da-GAL4/+*	F	1	SY	200	36	*da-GAL4>UAS-bmm vs. da-GAL4/+*	19.4	*p*<1e-10
	da-GAL4>UAS-bmm	F	1	SY	200	43			
	da-GAL4/+	F	2	SY	201	37	*da-GAL4>UAS-bmm vs. da-GAL4/+*	24.3	*p*<1e-10
	da-GAL4>UAS-bmm	F	2	SY	201	46			
	da-GAL4/+	F	3	SY	140	40	*da-GAL4>UAS-bmm vs. da-GAL4/+*	20.0	*p*=3.9e-05
	da-GAL4>UAS-bmm	F	3	SY	130	48			
	+/UAS-bmm	F	1	SY	116	48	*da-GAL4>UAS-bmm vs. +/UAS-bmm*	0	*p*=0.0006
Figure 6B	*da-GAL4/+*	M	1	SY	200	45	*da-GAL4>UAS-bmm vs. da-GAL4/+*	4.4	*p*<1e-10
	da-GAL4>UAS-bmm	M	1	SY	200	47			
	da-GAL4/+	M	2	SY	171	46	*da-GAL4>UAS-bmm vs. da-GAL4/+*	10.9	*p*=0.0284
	da-GAL4>UAS-bmm	M	2	SY	145	51			
	da-GAL4/+	M	3	SY	173	49	*da-GAL4>UAS-bmm vs. da-GAL4/+*	10.2	*p*<1e-10
	da-GAL4>UAS-bmm	M	3	SY	136	54			
	+/UAS-bmm	M	1	SY	139	59	*da-GAL4>UAS-bmm vs. +/UAS-bmm*	-8.5	*p*<1e-10
Figure 6C	*da-GS-GAL4>UAS-bmm*	F	1	EtOH	200	74	RU486 *vs.* EtOH	5.4	*p*=0.0025
		F	1	RU486	200	78			
	da-GS-GAL4>UAS-bmm	F	2	EtOH	199	69	RU486 *vs.* EtOH	4.3	*p*=0.0043
		F	2	RU486	200	72			
Figure 6D	*da-GS-GAL4>UAS-bmm*	M	1	EtOH	187	76	RU486 *vs.* EtOH	0	*ns*
		M	1	RU486	200	76			
	da-GS-GAL4>UAS-bmm	M	2	EtOH	201	69	RU486 *vs.* EtOH	0	*ns*
		M	2	RU486	200	69			
Figure 6E	*da-GS-GAL4/+*	F	1	EtOH	154	74	RU486 *vs.* EtOH	0	*ns*
		F	1	RU486	154	74			
	da-GS-GAL4/+	F	2	EtOH	200	72	RU486 *vs.* EtOH	0	*ns*
		F	2	RU486	200	72			
Figure 6F	*da-GS-GAL4/+*	M	1	EtOH	185	79	RU486 *vs.* EtOH	-3.8	*ns*
		M	1	RU486	183	76			
	da-GS-GAL4/+	M	2	EtOH	147	65	RU486 *vs.* EtOH	0	*ns*
		M	2	RU486	147	65			
